# Exploring the Socio-cultural Dynamics of Treatment Adherence amongst Females living with Pulmonary Tuberculosis in Karachi, Pakistan

**DOI:** 10.12669/pjms.41.9.11726

**Published:** 2025-09

**Authors:** Abeer Mian, Mehjabeen Musharraf, Lubna Ansari Baig

**Affiliations:** 1 Abeer Mian, PhD Population & Public Health, Senior Instructor, Community Health Sciences Department, The Aga Khan University, Karachi Pakistan; 2 Mehjabeen Musharraf, MSPH Instructor, Westman Immigrant Services, Manitoba, Canada; 3 Lubna Ansari Baig, Ph.D. Program Professor, Director, University College of Medicine and Dentistry, The University of Lahore, Lahore Pakistan

**Keywords:** Gender norms, Pulmonary Tuberculosis, Socio-cultural context, Treatment adherence

## Abstract

**Objective:**

To explore socio-cultural and grassroots factors influencing treatment non-adherence amongst females living with Pulmonary Tuberculosis (TB).

**Methodology:**

A qualitative Phenomenological design was adopted. Twelve in-depth interviews were conducted at a TB Clinic in Karachi (The Behbud Health Clinic) from February, 2020 to May, 2020 with participants including (i) females living with Pulmonary TB and (ii) healthcare providers engaged in TB service-provision. Interviews were semi-structured and conducted in-person. Data was analyzed inductively using thematic analysis.

**Results:**

Three themes contributing to treatment non-adherence emerged, which are: i) the burden of home-making with sub-themes of a culture of matriarchy, traditional household norms, and intended non-disclosure of TB status; ii) the journey of pursing treatment with subthemes of challenges in accessing diagnostic services, barriers in treatment adherence/continuation, unique treatment-related misconceptions; and iii) TB myths with subthemes emphasizing local rumors and an inherent lack of trust in public healthcare services.

**Conclusion:**

This study reveals important grassroots, socio-cultural and physical barriers to treatment adherence among women, including gender, social norms and treatment demands. Addressing these requires a holistic approach prioritizing community awareness with a focus on understanding day-to-day lived experiences of TB and building healthcare provider capacity to provide treatment services that are responsive and sensitive to these barriers.

## INTRODUCTION

Pulmonary Tuberculosis (TB), caused by Mycobacterium Tuberculosis, resurfaced in 2023 as the leading cause of infectious disease mortality, surpassing COVID-19.^1^ A record 8.2 million new cases were reported globally – the highest reported since 1995, with Asia bearing 56% of the burden.^2^ Within Asia, Pakistan ranks fifth amongst thirty high-burden TB countries. This disease is prevalent across various classifications, Category-I TB encompassing patients newly diagnosed with pulmonary TB; Category-II TB including patients with a history of TB treatment who have either relapsed, defaulted on treatment, or experienced treatment failure; and, increasingly, Multi-drug resistant TB. MDR-TB is characterized by *Mycobacterium tuberculosis* strains resistant to at least isoniazid and rifampicin, two of the most effective first-line anti-TB drugs.^2^ Annually, Pakistan reports 510,000 new cases, and it ranks fourth globally in the prevalence of multi-drug resistant TB.^2^

A review of literature attributes this burden to squatter conditions, malnutrition, unsupervised treatment and poor follow-up and referral.^3^-^5^ In light of this, the National TB Control Program (NTP) in Pakistan has made significant efforts in controlling TB, with the expansion of Directly Observed Treatment, Short Course (DOTS), increased case detection and a strengthened laboratory network.^6^,^7^ However, challenges such as drug resistance, stigma, limited infrastructure and more recently deteriorating political will & interest in the TB agenda persist.^7^

Pulmonary TB continues to disproportionately affect females in Pakistan. This is particularly concerning given women’s heightened risk of exposure as primary care-givers and underpins a disconnect between existing policy, programming, and realities faced by affected individuals. This study aims to bridge this gap by delving deeper into the lived experiences of TB amongst women at the grassroots level. By understanding how TB treatment behaviors and knowledge are shaped, learned, and transmitted within a specific context, this study seeks to gain deeper insight to contextualize TB protocols relative to the specific sensitivities and struggles faced by women living with TB. Thus, this research will explore socio-cultural and grassroots factors influencing treatment non-adherence amongst females living with Pulmonary TB. Insights on unique barriers and facilitators to care-seeking and adherence will be derived from the voices, views, and first-hand experiences of female TB patients, their caregivers, and healthcare providers.

## METHODOLOGY

This study was conducted within a pulmonary TB center located in a low-middle income, urban community in Karachi. A qualitative study using the phenomenological approach was used to explore the lived experiences and perspectives of female Pulmonary TB clients & healthcare providers regarding treatment adherence.

### Ethical Approval:

This study received ethical approval from the Institutional Review Board of Jinnah Sindh Medical University for the year 2019-2020 on August, 29 2019 (Reference No: JSMU/IRB/2019/-188).

Theoretical saturation was used for sampling. Guidelines in the literature indicate that a phenomenological approach should involve sustained engagement with a smaller number of subjects.^8^ Further studies suggest this small number as starting with six participants.^9^ This was used to set the minimum anticipated number of interviews for this study. Additional interviews were then conducted until no new themes emerged.

Purposive sampling was applied to select participants in two categories; female persons living with Pulmonary TB; and healthcare providers engaged in TB service provision. The eligibility criteria for women living with TB included (i) females aged 18 years and above who were diagnosed with pulmonary TB (through chest X-ray and/or sputum testing), (ii) categorized as either relapse cases or treatment after default cases, and (iii) receiving treatment for at least six months at the center. This information for client category and treatment was obtained through reviewing medical record files (with permission from the Senior Medical Officer of the center). Those (i) diagnosed with childhood TB, (ii) extra-pulmonary TB, and (iii) refusing to provide consent were excluded. The eligibility criteria for healthcare providers included those (i) working for at least two consecutive years at the TB center and (ii) with more than eight years of education. Those who (i) intended to leave the center within one year and (ii) refused to provide consent, were excluded.

Twelve in-depth interviews (IDIs) were conducted in total (as shown in Table-I) in local Urdu language. The interview guides for female Pulmonary TB clients and healthcare providers were semi-structured to explore various facets of TB treatment and compliance. For women living with TB, key areas and probes included their initial response to diagnosis, daily treatment challenges and perceived solutions, the role of their social support networks, and specific factors and barriers to medication adherence. The guide for the Healthcare providers was focused on questioning on methods of client support, the struggles they encountered in facilitating treatment, their observations of client cooperation and compliance challenges, the perceived commonality of these challenges among other clients, their strategies for resolving compliance issues, and the socio-cultural factors they believed influenced client motivation.

**Table-I:** Participants interviewed.

Category	Participant Type	In-depth Interviews
Female persons living with Pulmonary TB	Category I & II TB case	5
	Multi-drug Resistant case	1
TB-related healthcare providers	TB Treatment Facilitator	2
	TB Treatment Supporter	1
	Senior Medical Officer	1
	TB Clinic Chairperson	1
	TB DOTS Field Supporter	1

All IDIs were held in-person, in a private room in the center and audio-recorded after obtaining consent. Data was analyzed inductively using thematic analysis. Transcripts were coded inductively, followed by axial coding to identify subthemes. Selective coding was applied to refine these into final themes. Two reviewers coded the transcripts separately; coding was finalized through consensus. Member checking was done to ensure confirmability by reviewing the transcripts with participants.

To ensure rigor of this qualitative study, we adopted three key strategies. To establish credibility and ensure our findings genuinely reflected participant perspectives, a meticulous interviewing process was applied. Pilot interviews were conducted with participants from each category to verify that our guides were clear, easy to understand, and both contextually and culturally appropriate, with modifications as required.

Following initial transcript analysis, a trustworthiness check was performed with participants at the center. This step confirmed that the interpretations and findings accurately represented what participants expressed and intended. Furthermore, to ensure the dependability of the study findings (the ability to replicate the inquiry with similar results), a rich description of methods was provided. This included a detailed account of the research context, participants and tools.

We further strengthened the confirmability through a researcher triangulation strategy. As part of this strategy, two independent reviewers coded the interview transcripts. All codes on each transcript were then cross-matched, and any areas of divergence in coding and theme extraction were shared and discussed with a third neutral reviewer for finalization.

## RESULTS

**Fig.1:**
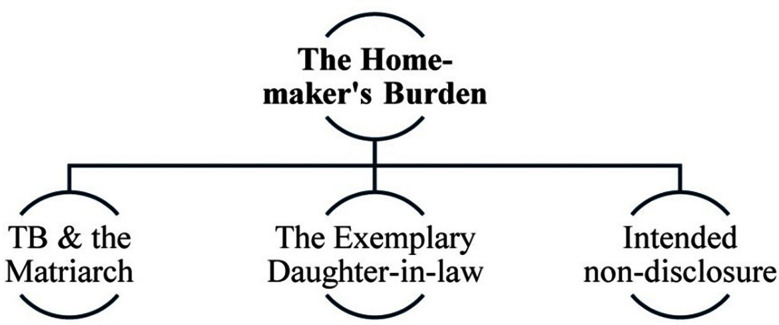
Theme-1 & subthemes - The Home-maker’s Burden.

**Fig.2:**
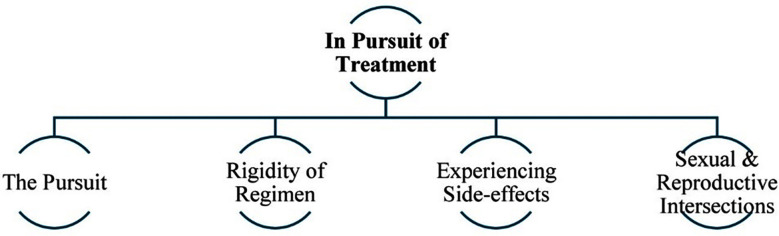
Theme-2 & subthemes - In Pursuit of Treatment.

**Fig.3:**
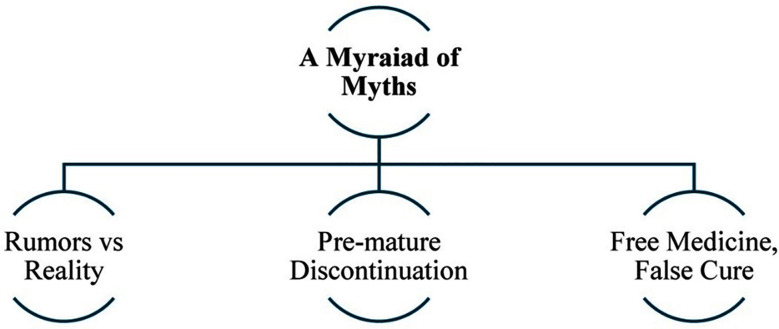
Theme-3 & subthemes - A Myriad of Myths.

This study explores reasons for treatment non-adherence amongst females living with TB despite the wide-scale availability of free-of-cost treatment. Three key themes with ten corresponding sub-themes (three in the first theme, four in the second and three in the third) emerged from the analysis as shown in Fig.1-3.

### Theme-1: The Home-maker’s Burden:

The study revealed that treatment adherence is heavily influenced by complex socio-cultural factors and practices that shape participants’ ability, agency and motivation to adhere. (as shown in Fig.1).

### TB & the Matriarch:

The burden of homemaking is widely shared among Pakistani women. Their physical and emotional labor is constantly expended to maintain a clean household, cook meals, and care for children at the behest of household members. Their rights and needs are influenced by household dynamics particularly the nature of the matriarch. An educated mother-in-law, or one who has personally experienced TB in the past, would be more emphatic to the suffering and severity of the disease and predisposed to encouraging her daughter-in-law to seek treatment, as described by a Treatment Facilitator;

*“Her [a patient’s] mother-in-law used to tell her when she was having difficulty breathing and coughing to get TB tests done from this center. She brought her here to get the X-ray done. When we found out it was TB, she kept telling me treat my daughter-in-law. Start her treatment. She was the only one and she came with the patient every time.”*

However, if the matriarch lacks knowledge of TB, she will likely dismiss her daughter-in-law’s suffering, discourage and disapprove of seeking treatment; rendering her daughter-in-law’s status to what a Treatment Facilitator referred to as “nokrani (*servant)* of the house”.

*“Actually, the in-laws wed the girl into their home with a lot of celebration but she is given no value once there and she becomes a servant expected to work all the time in the house. So when they don’t give a daughter-in-law izzat (respect), her status lowers even more when she is sick.”*

### The Exemplary Daughter-in-Law:

The study participants reported instances where in-laws accused them of feigning illness to make excuses to “rest” and “laze around”. They were reportedly mocked and criticized for failing to contribute to the household and were compared, unfavorably, to other ‘dutiful’ and ‘normal (i.e. healthy)’ daughters-in-law in the neighborhood. Because their illness was perceived to be ‘fake’, household decision-makers failed to address or acknowledge their need to seek care or consistently continue treatment. The presence of TB therefore plays an important role in the social construction of the exemplary daughter-in-law, as narrated by a TB field supporter;

*“It is the duty of a woman to care for her elders and be good at housework, so there is always competition on which daughter-in-law manages her household best. If a daughter-in-law is sick, she is unable to do work, she is considered lazy and gets taunted for doing less than the other daughters-in-law. She is compared to them. Her mother-in-law would say to her to stop pretending, do work like all the other daughters-in-law.”*

Many participants therefore reported prioritizing household chores over their treatment to avoid being taunted. This led to missing medication doses at the prescribed time, as they were pre-occupied with completing household tasks to the satisfaction of their in-laws, as stated;

*“If a woman is unable to complete her housework, her in-laws look at her with anger and scold her…she then gives no importance to her treatment and says ‘leave the medication’ right now I have to get this work done… They would neglect themselves and put their treatment aside rather than having to listen to constant taunts.”*

Alongside missing medication doses, women face challenges leading to delayed follow-up visits & critical testing for TB treatment. In-laws, in many cases, refuse to assist in chores and childcare, leaving women to complete these tasks before seeking medical care. This leads to missed appointments and most importantly delayed medication refills and crucial tests, impacting decisions on next steps in treatment (including changes in medication doses and sequence), as a participant recall;

*“They [female living with TB] mostly say I have no one, my in-laws say take care of your own child, take them with you. Or my husband says he has to go work and I can’t leave my child. So, she has to take care of all things before she steps out of the house to collect medicine. Mostly, by the time they get free, it’s too late and they have to wait till the next clinic.”*

### Intended Non-disclosure:

Two participants concealed their TB diagnosis from their families due to fear of being taunted, facing social stigma and isolation. They expressed that within their households, TB is often misunderstood and viewed in the paradigm of its historical roots – the Sanitorium – a facility for quarantine and permanent isolation. The symbol of the Sanitorium is still strongly resonant in the ways that participants explain how their families and elders suffered from TB, i.e., by being kept in complete isolation. Stemming from this is a fear of being separated (particularly from young children), that led participants to prioritize their family’s acceptance over disclosing their TB status and timely seeking care, as highlighted by TB Clinic Chairperson;

*“Having TB is a big social taboo. Even bigger for a woman. People don’t even know it can be treated fully. All they know is that this disease will spread, so… they will actually throw a woman with disease out like, she is poisonous. Even before they can think of treatment, she is already considered a danger.”*

Similarly, participants expressed worry over being blamed for spreading Pulmonary TB to family members and the community, despite potentially contracting the disease from someone within their own household, in the first place. This highlights a fear of blame and guilt perceived to be potentially borne by participants, compelling them to hide their TB status. Most then seek TB treatment discreetly which affects their ability to adhere to treatment in two ways. First, it prevents timely medication intake, in that, participants are unable to take their medicine at the prescribed time (30 minutes before breakfast, on an empty stomach) simply due to the presence of other family members. Second, it limits the supportive supervision from household members necessary for ensuring correct and consistent medication usage. Additionally, the inability to inform family members about weekly follow-up visits leads to missed appointments and increased likelihood of non-adherence, with eventual onset of drug resistant strains, as narrated by a client living with MDR TB;

*“I wouldn’t take the medicine in front of everyone, so I could not have it at breakfast. We all have breakfast together in my houses so I took it with me to work…If I told them they would not let me take medicine or do my treatment. When I wanted to go to Doctor Sahib at the center, I knew they would ask where I was going and send my younger brother-in-law with me. I couldn’t leave the house alone. My medicine used to finish and so my cough didn’t get better. “*

### Theme-2: In Pursuit of Treatment:

Beyond the social household dynamic, the process of accessing and subsequently completing TB treatment is an exhausting pursuit. This theme with its corresponding sub-themes shown in Fig.2, weaves together the challenges of the journey of this pursuit.

### The Pursuit:

Underdiagnosis and misdiagnosis of TB led participants to delay seeking treatment for their ‘misunderstood’ symptoms. When they finally decided to seek medical attention due to increasing severity of symptoms experienced, they reported facing major inconveniences with accessing facilities (before they came to know about and sought treatment at the study clinic). These included long waiting times (often in the heat), non-availability of doctors, absence of critical TB testing services such as sputum testing, all of which worsened their symptoms of fatigue, fever and overall function as relayed, in part, by a client living with TB;

“*I didn’t feel like waiting in those lines. I can’t even breathe and my body hurts. The fans were not even working in the room where we waited. It happened many times that I just felt like leaving from there and forgetting that I was sick.”*

Participants further reported that they had to run between several centers in different and usually distant parts of the city for diagnostic tests including (i) Chest X-ray examinations, various blood & stool tests and Sputum testing (either AFB Smear test or GeneXpert testing). They received these test reports from one center, then had to go to another to consult Doctors/Specialists to receive a definitive diagnosis, treatment plan and prescription. In an effort to minimize treatment costs, a third layer was added by travelling to a third center/ pharmacy for discounted medication. Participants were also willing to travel longer distances to access centers offering affordable or free services, particularly for Chest X-rays and GeneXpert sputum testing. A participant describes her trek as;

“*There was a lot of khuwaari (nuisance) in having to run around. Someone would say go to X hospital and get tests done then go to Y hospital as they do it for free there. I would leave in the afternoon and the whole evening was spent going from one place to another. I spent a lot of days doing this running before we found out it was TB. I had to take my children. No one takes care of them. After some time, we were fed up.”*

### Rigidity of Regimen:

Overcoming the initial challenges of accessing TB treatment is just the beginning. The treatment itself is demanding and requires strict adherence, caution and consistency over a prolonged period of at least six months. Medication needs to be taken every single day in the prescribed dose, exactly half an hour before breakfast, on an empty stomach. This is an essential pre-requisite to effective TB treatment, particularly during the initial Intensive Phase, consisting of a rigid two-month combined heavy-dose medication regime of Isoniazid (INH), Pyrazinamide (PZA), Ethambutol (EMB) and Rifampin (RIF) medication. This is followed by the Continuation Phase, consisting of at least four months of an INH-RIF regimen. Skipping a single dose during this phase reduces treatment effectiveness, prolonging its subsequent duration. Thus, medication adherence is perceived as a physically & mentally challenging task that requires swallowing large, hard pills in an ‘unforgiving’ sequence, as narrated;

*“Patients get fatigued easily. It is not easy to wake up every morning and take heavy and big tablets first thing. It’s not only one, it’s four. For the first two months, there is no question that the patient can miss even one day’s medication. when it’s not nice to look at the medicine, how will one feel when having to swallow it every morning.”*

Participants equated the physical attributes of TB medication as a demotivating reminder of their illness and the long and difficult journey of treatment ahead. The pills then become a symbol reflecting their perceived bad luck, hardships and discomfort in relation to living with TB, as expressed;

“*I feel disgust when I look at the medicine. It has to be eaten every morning. It is very hard for me to swallow this medicine; it hurts my throat. That’s why I just left it sometimes. It was very difficult. as, I would not feel like eating in my illness but had to take this.”*

Moreover, as per the TB treatment protocol of the selected clinic, follow-up conducted on a weekly basis involves a time-consuming sequence of; (i) Chest X-ray examination, (ii) sputum sample submission & testing, (iii) weight & vitals assessments, (iv) detailed client reporting & forms (TB01 & TB02) and finally (v) changes to client’s treatment prescription. Despite recognizing its importance, many participants stated it was simply not practical to adhere to this rigorous schedule due to time constraints and transport costs (going to the center every week). A Senior Medical Officer expressed;

*“TB treatment is very demanding for the patient to become regular and strict with taking their medication. We[Doctors] understand this treatment is not easy. That’s why we try to counsel a patient to prepare them for what they will go through in the next six to eight months, to make them receptive and ready for this long treatment.”*

### Experiencing Side-effects:

While rigidity of treatment regi-men significantly reduces likelihood of treatment adher-ence and continuation, it is compounded by the issue of side-effects experienced by study participants.

The medication side effects most commonly reported in this study were; excessive itching, gastrointestinal issues, joint pain, skin rashes and dizziness. Dizziness and stomach problems significantly affected adherence. Participants reported skipping medication doses to alleviate these side-effects, without prior medical consultation. One client made an uninformed decision to reduce her dosage from three tablets per day to every alternate day, to mitigate diarrhea, eventually defaulting on treatment;

*“Taking medicine early in the morning without eating anything makes me feel very dizzy. Even walking down the stairs is difficult. My head spins and I still have to stand on a burner to make breakfast, which is dangerous and hard, so many times I left my medication at that time and forgot to have it later..so I didn’t complete my treatment at that time.”*

Some participants managed side effects either by abandoning or taking additional medication, prescribed after consultation with a doctor. While continuing their TB regimen, they found the extra medication an added ‘nuisance [museebat]’ to the already demanding task of adhering to taking the requisite hard pills. Rather than a cure, the treatment regimen was viewed, by some, as a source of further health complications, undermining their faith in treatment effectiveness and tiring their will to adhere, as expressed;

*“Patients often complain when they start medicine. Many experience skin, stomach and joint pain issues. They have to take added medicine for that and so lose trust in treatment because they are taking so much medicine and have to keep taking more for its side-effects.”*

### Sexual & Reproductive Intersections:

The rigid treatment protocol and uncomfortable side effects, combined with participants’ worries about their sexual and reproductive health, further increases the likelihood of non-adherence. Within this study, sexual and reproductive intersections are explored through perceptions of women living with TB as their ability to (i) engage in physical intimacy during treatment, and (ii) conceive while on treatment regimen.

Family members, particularly husbands, reportedly perceive TB medication as negatively impacting sexual performance, leading to increased tension, anger and conflict within spousal relationships. This perception compounded by religious expectations that women should prioritize their husbands’ needs, socially burdens women living with TB, leading them to deliberately avoid treatment medication, as told by a client;

*“My husband would also be upset with me. He kept saying the medicine they give you is making you weak. How can we go on like this…our men don’t like all this. Even God gets angry with you if you refuse [one’s husband] like this.”*

Many participants experienced missed menstrual cycles during the intensive treatment phase, a temporary side-effect. This reportedly caused significant distress as it was perceived as a threat to fertility and emerged a grave concern for women whose societal value and respect within their household was tied to their ability to bear children. In some cases, participants reported being pressured by family members to discontinue their medication to increase chances of pregnancy. One participant shared that, eight to nine months into her marriage, her mother-in-law began pressuring her to get pregnant. When she revealed she missed two menstrual cycles while on TB medication, she narrated;

*“My mother-in-law scolded me and told me this is enough, you will not have this medicine, it is cursed, it is stopping you from giving your husband children, to the family and you keep on taking it.”*

Decisions/coercion to stop adhering to medication on the grounds of infertility are often reinforced in a religious context. Whereby, missed menstrual cycles induced by treatment are perceived as a deliberate act of rejecting supposedly divine blessings, particularly the ability to have multiple children. Therefore, clients’ inclination to adhere to treatment is heavily (mostly adversely) influenced by surrounding socio-cultural and religious beliefs, as expressed;

*“Patients used to take medicine in their houses. But one or two times, women complained they were missing their periods. I went back to their homes and explained it was a side-effect that would go and don’t stop taking your medication. There was one patient with this period issue…when I went back to her house after that, they didn’t even open the door for me… she did not even answer to take the medicine.”*

Conversely, religious discourse has been adapted by clinic staff to encourage treatment adherence and enhance conception and fertility prospects. The underlying principle used by participating staff members was that God does not impose burdens beyond one’s self-capacity. Inserted into the context of TB, this meant that a weakened body, susceptible to fatigue, medication side-effects, and exhaustion, necessitated adherence to treatment for recovery and subsequent family planning, as explained;

*“Yes it is true that TB medication will not stop you from getting pregnant. But it does affect the woman’s body. She has to keep a child in her body for nine months…When you have TB yourself, and you don’t even feel like eating, you have no nourishment to give to a child. We try to say that God does not want a sick person to suffer more, so complete treatment and then conceive.”*

### Theme-3: A Myriad of Myths:

The perceived impact of TB treatment on female reproductive health is merely one facet of the broader spectrum of TB-related misconceptions. This theme, with corresponding sub-themes (shown in Fig.3), takes a deep dive into deeply-rooted misconceptions and how they influence TB treatment behaviors amongst participants.

### Rumors vs Reality:

It was evident from interviews, that participants often relied on rumors rather than scientific evidence to understand TB, contributing to delayed treat-ment and low adherence. One participant stated she be-lieved her TB was caused by stress and grief following her father’s loss. Existing beliefs, knowledge, and attitudes towards TB, amongst participants, seem to be widely characterized by uncertainty and misunderstanding which instigates fear and shame rather than hope for a cure, as a Senior Medical Officer mentions;

*“Awareness is missing on TB. There is no knowledge of proper treatment of TB, it’s not a fatal disease. Patients focus on the dangerous aspects of it rather than the cure that God has provided ..Just in the same way, that people do not mention the beauty of heaven, but they scare you from the fire of hell.”*

To add to this, TB was also described as a fatal disease without any cure, by a woman living with TB;

“*My cousin had told me that TB is a fatal illness. That’s why I wasn’t even sure about the treatment and whether it would be helpful. I told myself that I was going to die because of it.”*

### Pre-mature Discontinuation:

Another common misconception was that medication could be discontinued as soon as symptoms disappeared. TB symptoms usually subside within the first two months of treatment (Intensive Phase). A Treatment Supporter reported that the disappearing of symptoms led at least four women living with TB, to deceivingly assume they were fully cured of TB and discontinue treatment without due medical consultation, leading to one of them defaulting to a higher category of TB, as described;

*“Most patients start feeling better after taking the medicine for two months. their fever subsides, they put on weight and the pain goes away. So, some of them think they don’t need treatment anymore and don’t inform us or come for follow-up. After months, they will come back and say now we have worse symptoms.”*

### Free Medicine, False Cure:

Moreover, the effectiveness of TB treatment is often questioned, particularly when it is provided free of charge. Participants reported that their family members doubted the efficacy of medication solely due to it being provided at no cost. In that, if it were inexpensive or free of charge then, as described in the words of a client, according to her father-in-law, it was “spoilt/ expired” and “had to be gotten rid of, so it is[was]given away for free”. This perception reflects a common belief that expensive medications are more effective. Similarly, two participants shared that individuals within their neighborhoods believed that taking TB medication reduced breast milk production, compromised its purity and harmed infants, which discourages adherence and more broadly even seeking or initiating treatment, as elaborated by a Treatment Facilitator;

*“Someone from the community will always make these statements, whether a TB patient can breastfeed her child or not. For that if their understanding is that she can’t, then treatment will not even be considered… We hear many questions like this. One woman said that the amount of her breastmilk was being reduced right after taking the medicine.”*

This reflects the conviction and persistence with which TB disease and treatment misconceptions are held. Providers emphasized the need for comprehensive and strategically-timed individual counselling sessions to (i) address and dispel these myths, (ii) reinforce the importance of treatment adherence, (iii) educate clients on side-effects and, (iv) provide frequent physical, social and emotional support throughout the treatment journey, as stated;

*“There’s a big counseling role in treatment. You need to explain each and every part of treatment, as if you are speaking to a child. That no your chances of getting pregnant will not be affected, you can still breastfeed. Counseling is an ongoing process.. which won’t happen over one sitting, rather over many months.”*

## DISCUSSION

This study highlights the impact of domestic responsibilities and social expectations on adherence to treatment amongst women living with Pulmonary TB. Supporting literature from South Asia and Sub-Saharan Africa indicate that women disproportionately experience unsuccessful treatment outcomes due to social and marital repercussions of living with disease. A study in India reported that 10% of women with Pulmonary TB experienced divorce (especially among younger spouses), 25% faced isolation and discrimination within their homes, 18% were rejected by their husbands and in-laws, and 40% were deemed unfit for marriage.10-12

The literature also reveals a gendered disparity in care expectations, with males expecting care from their wives while TB-affected wives rarely received adequate support.11 Similarly, a study in Zambia investigating treatment adherence reported that males were older, more educated and more likely to adhere to full course of treatment than females. 39.1% of females in the study discontinued treatment within the first two months citing household issues and stigma as barriers of adherence.13

This study also highlights unique misconceptions about TB, including its impact on fertility, conception and sexual intimacy. These misconceptions remain rooted in low literacy rates and limited health education which delay care-seeking decisions relating to diagnosis and treatment.13 Studies from Muslim-majority countries including Ghana and Somalia document similar findings where religious and cultural beliefs impede timely care-seeking and treatment adherence, specifically in relation to married women and young mothers.14-17

### Limitations:

This qualitative study, while providing valuable insights, has limitations. In this specific research, social desirability bias18 could have potentially influenced participant responses, making them hesitant to admit treatment non-adherence due to fear of stigma, judgment, or potential consequences, leading to under-reporting missed doses or incomplete treatment. To address this, the researcher, through prolonged engagement, attempted to build rapport with participants through regular clinic visits to create a trusting and supportive environment for participants to share their experiences comfortably and candidly.

## CONCLUSION

This study highlights significant socio-cultural and physical barriers to TB treatment adherence amongst women including social norms, stigma, the demanding nature of treatment and associated misconceptions. Ad-dressing these challenges necessitates a holistic approach that considers the unique socio-cultural factors influencing TB treatment adherence amongst women living with TB in different urban communities. By highlighting grassroots voices & experiences, the focus should remain on empowering communities to understand and address TB within their own context.

### Recommendations:

Future research should prioritize the intimately lived experiences of TB (amongst males & females), to develop evidence-based health education and promotion strategies to improve timely care-seeking and treatment adherence behaviors. To effectively address the unique barriers identified in this study, it is essential to provide healthcare professionals with capacity-building opportunities, particularly in counseling skills. This is essential given the high prevalence of Pulmonary TB in Pakistan.
